# Improve the quality of end-of-life in cancer patients using social representations of nutrition

**DOI:** 10.3389/fonc.2024.1386953

**Published:** 2025-01-03

**Authors:** Lucia Inmaculada Llinares-Insa, Encarna Chisbert-Alapont, María Amparo Benedito-Monleón, Pilar González-Navarro

**Affiliations:** ^1^ Facultat de Psicología, Departamento de Psicología Social, Universitat de València, Valencia, Spain; ^2^ Servici d’Hematología, Hospital Universitari I Politècnic La Fe, Valencia, Spain; ^3^ Facultat de Psicología, Departamento de Psicología Social, Instituto de Investigación en Psicología de los RRHH, Desarrollo Organizacional y Calidad de Vida Laboral (IDOCAL), Universitat de València, Valencia, Spain

**Keywords:** cancer, nutrition, palliative care, social representation, end-of-life

## Abstract

**Introduction:**

The problems related to nutrition generate great concern in palliative cancer patients and their caregivers. Literature has analyzed the psychological and social problems that nutrition causes. From patient-centered orientation, there are protocols for nutritional care. This psycho-health information is a source of discomfort among patients and families and its absence generates many problems related to nutrition with negative clinical repercussions. Thus, this study aims to analyze the concept of nutrition in advanced cancer patients with palliative care and their caregivers. Furthermore, given that food has an important cultural component as important as it is nutrition, the sociocultural influence on the social representation of nutrition will also be analyzed.

**Methods:**

The study design was qualitative and cross-sectional with a recruitment period lasting 18 months. After the informed consent, a sample of advanced cancer patients (N=57) and their caregivers (N=57) were interviewed individually. The data was analyzed using content analysis and descriptive analysis. The existence of statistical differences between groups (patients and caregivers) was tested by Chi-Square statistics.

**Results:**

Results showed that the perception of nutrition was structured in five categories: health/survival/life (e.g. “healthy”), social relationship (e.g. “intimate relationship”), care (e.g. “care role”), foodstuff (e.g. “vegetables”), and others (“anything”). There were significant differences (p-Value>0.05) in the uses of nutrition between patients and their caregivers and caregivers had higher scores. Then, nutrition was perceived as an act of care and, therefore, patients were expected to strive to eat despite the loss of appetite.

**Discussion:**

This study is one of the first to explore the perception of nutrition emphasize the insufficient consideration of patients’ and caregivers’ needs and perceptions regarding food, as well as the significance of this knowledge in patient-centered care approaches. In this way, it could intervene by first understanding the behavior concerning food and, secondly, redirecting the behavior if it is harmful to the patient or family relationships regarding the health care and well-being of patients with advanced cancer with palliative care.

## 1 Introduction

Cancer is a critical illness, and it is now rapidly increasing. In Spain, it is one of the leading causes of death. When a patient faces a terminal health condition, palliative care becomes a crucial type of care for the end-of-life stage (1). Palliative care aims to improve the quality of life and well-being of the patient and their families (2). Health professionals are key players because they are specifically trained to analyze and intervene on any concerns they may have, care for their needs, and carry out interventions to reduce patient and caregiver distress (3). One of these problems is concerned with feeding and nutrition since nutrition and feeding are key components of patient care (4).

Many researchers analyze nutrition in palliative care patients and clinical guidelines have been proposed to guide the actions of healthcare personnel (5). These protocol practices concern nutritional care, but the needs, experiences, and perceptions of patients and their caregivers are not included, and these are core for ‘patient-centered’ orientation (6). This topic is a gap in the specialized oncology sector, and, following Leoliger et al., (7), it is necessary to improve research on it. In this sense, this paper analyzes the social representation of patients and their caregivers regarding nutrition. Thus, from a patient-centered approach, the aim is also to integrate the results into oncology health practice and routine care as well.

One cause of distress between patients and family is food because of palliative cancer patients have nutritional problems (8). Patients with palliative cancer have gastrointestinal problems, with symptoms such as nausea, vomiting, diarrhea or constipation, premature satiety, etc. (9). This situation can generate nutritional deficiencies because of the lack of appetite and the decrease in weight that occurs in high percentages, affecting the patient both physically and psychologically. Moreover, malnutrition is frequent in some types of cancer, for example HNC (10) and a significant problem for some palliative cancer patients and generates negative clinical repercussions (see 11).

One line of research on palliative care analyzes adequate nutritional interventions because they can influence patient clinical outcomes and/or promote well-being, comfort, and quality of life (12). On the other side, the negative hand is also considered an indicator of survival and proximity to death (13). Some studies report that 20% of mortality in terminal cancer patients is caused by malnutrition (14). However, the relationship between nutrition and cancer is complex. Researchers are analyzing, for example, good and bad foods and the interaction between certain foods and certain medicines (e.g. 15). Another line of research is analyzing which is the ideal diet. Researchers are also analyzing appropriate nutritional strategies and making nutritional recommendations for palliative care diet and nutrition (16). Nutritional screening and assessment are additional aspects being analyzed by researchers as well as early identification (17). There are studies over food enjoyment and how to minimize the discomfort produced by the ingestion of food (18).

Other studies have examined the significance of nutrition for cancer survivors in the post-treatment stage (19). In survivors, efforts have also been made to understand and describe the barriers to healthy eating (20) and concluded that there is a need for more cancer-specific nutritional advice and support. However, in Spanish hospital and home care there are not control of what patients eat or their appetite. Then, health professionals do not know the quantity and variety of foods PCP. There are also more novel attempts in the literature such as that of Qi et al. (21) who have proposed an integrated rehabilitation model of exercise psychology and nutrition based on mobile health and virtual reality in people with cancer.

On the other hand, in the literature on nutrition and cancer, we find numerous recommendations based on scientific evidence that results from the consensus of healthcare personnel. However, there are often disparities in nutrition information and advice provided by healthcare providers that can become contradictory (6). Thus, patients and their caregivers express great interest in nutrition and obtaining information, sometimes contrary, from health services (see 22). Then, some patients disregard nutritional recommendations which can generate problems between themselves and their family members (23). In this type of situation, it would be valuable to know the social representation behind it all for palliative cancer patient and their caregivers to reduce anxiety. Moreover, healthcare personnel should assist patients and caregivers to clarify misconceptions about food and reduce anxiety (3) about nutritional problems. We do not know of other studies that investigate the social representations of nutrition or studies about this meaning towards feeding and food in this phase of the disease. This knowledge will allow professionals to know the perspective of the patient and their caregiver and understand their behavior to adapt the care to their needs.

Social representations are interpretations of common sense that guide behavior; they play a role in the decoding and interpretation of reality and predispose to action. They are very important because they are at the basis of the behavior patterns of both patients and caregivers. These can positively or negatively affect behavior. Some authors report that they tend to have a negative effect on the process of adjustment to the disease and, therefore, should be studied (24). However, we do not know of other studies that investigate the social representation in this phase of the disease. In this sense, there is a gap regarding the social representation associated with nutrition.

We based our study on palliative and end-of-life care of Gwyther et al. (2) and ‘patient-centered’ approaches (6). This knowledge will allow professionals to comprehend the perspective of the patient and their caregiver and understand their behavior, allowing them to adapt the care to their needs. The knowledge of social representation of nutrition is necessary.

With respect to social representations, we use the theory of Moscovici (25). This theory highlights the importance of social knowledge for the change of people’s behavior. And, for its understanding and analysis, we start from the study of Corrales et al. (26) que analyzes theoretical levels of the perception of nutrition when patients are at the end of their life. The researchers propose three categories of perception: nutrition as survival and life and its relationship to a healthy body, nutrition as a social relationship and to get pleasure, and nutrition as care and nutrition of family. Some researchers affirm that it is one of the most important aspects of survival (13). So, the lack of hunger is related with illness or death. Moreover, nutrition is one of the most complex aspects of life. In addition, numerous studies show that eating in the company of one or more people has a positive effect on food intake (8). Specifically, nutrition and food habits have a relational dimension. Then, nutrition seems to play a supportive role (27). Currently, food is considered an important source of affection. That is why nutrition can be a channel of affection when cancer patients continue with food habits, but it can also turn into disapproval when s/he does not continue. In this sense, nutrition is perceived how a special form of care and protection of family. Families and patients can consider it as basic care but also as medical treatment. The doctor is often afraid that the patient’s lack of food will make his/her health worse. However, some researchers believe that at the end of life, nutrition does not benefit the patient (28). They all agree that in this palliative stage, discomfort should be minimized, and welfare should be increased (see 23). However, that nutrition is more cultural than nutritional (29). So, the perception of nutrition is similar among all people of a single culture whether they are doctors, nurses, patients, or family members, …; it reflects socio-cultural influences. In this sense, the social representation of nutrition has a socially marked central core and a peripheral system that is individual and determined by the experiences and histories of each person (30). We are interested to identify the social representation of patients and caregivers to increase information on palliative care.

## 2 Method

### 2.1 Study design

The study design was cross-sectional and of a qualitative research design. Patients and their family caregivers were recruited from palliative care units of public and private hospitals for 18 months. Patients (sample 1) and their family caregivers (sample 2) were identified services and were contacted and were invited to participate in this study. The sampling procedure used to sample 1 and 2 was incidental purposive sampling. We used this sampling technique because these a group with special characteristics for their vulnerability and difficult access. Data were collected through face-to-face interviews at the patient’s home by a trained interviewer. The interviews with the patient and his caregiver were performed separately without the presence of other people to ensure spontaneity in the responses. The interviews were recorded prior to consent and transcribed later. The responses were confirmed by the informants through feedback to the interviewer. The researchers stressed that anonymity and confidentiality were guaranteed, that there were no right or wrong answers, and that participants should answer the questions as honestly as possible. The study was approved by the Ethics Committee of the Valencian Institute of Oncology (protocol number 1/2011).

### 2.2 Participants

The total sample was 114 (patients and their family caregivers) between 27 and 96 years of age (M=64.35; SD=15.67). Sixty-two percent are women and 61.4 percent are married or living with a partner. 65.8% have basic education and only 13.2% have higher education (see **Table 1**). Data was collected through face-to-face interviews by a trained interviewer at the patient’s home. Inclusion criteria were: had a verified cancer diagnosis and they were at the end-of-life stage, aged above 18 years, physically and mentally able to participate, their functional level was 2 or 3 on the Eastern Cooperative Oncology Group (ECOG) scale, and without cognitive alterations, they have a family caregiver and both lived together, and they provided written informed consent. Parenteral nutrition was an exclusion criterion. For both groups, we considered that cognitive or mental illness as well as difficulties in communicating are exclusion criteria.

**Table 1:** Description of two samples.

	Spanish palliative cancer patients (N=57)			Caregivers (N=57)			
	%	Mean	SD	%	Mean	SD	%
Men	43.9			31.6			38.5
Women	56.1			68.4			61.5
Age		70.68	12,29		58.02	16.0	
Marital status							
Single	3.5			14.0			50.8
married	61.4			78.9			40.8
Separated/divorced	5.3			1.8			5.4
widower	29.8			5.3			3.1
Highest Educational Level Attained							
Elementary School	77.2			54.4			19.2
Professional Training/Bachelor	10.5			31.6			23.8
Higher Education	12.3			14.0			56.9
Kind of cancer							
Breast	22.8						
Prostate	17.5						
Lung	14.0						
Others	45,7						
ECOG							
2	49.1						
3	50.9						
Relationship							
Spouse							49.1
Son/daughter							42.1
Sibling							5.3
Parents							1.8
Friends							1.8

SD, Standard Desviation; N, Sample; ECOG, Eastern Cooperative Oncology Group.

### 2.3 Instruments

To analyze the perception of nutrition we used a face-to-face semi-structured interview following the steps indicated by Valles (31). The first step to make the interview was a systematic review of the literature. The thematic lines identified in the review were the perception of nutrition, nutrition as a form of care, nutrition as a basis for survival, and nutrition and its benefits and uses. Thus, the interview covered all topics. Some questions were open-ended, and others were closed following the recommendation of Jamsjed (32) and Turner (33). The first part included the participant’s socio-demographic information. Then, we demanded free associated words (“When you hear the word nutrition, tell me what other words come to mind”), a question about benefits and uses (e.g. “Please finish the following sentence: Thanks to food, it is possible to achieve…), care (“When you are sick tell me 2-3 things that your relatives do to take care of you”), and survival (“Do you think that nutrition will influence in some way the course of your illness, that is, if you eat will you live longer? Why?”). Participants were asked to answer the questions spontaneously.

### 2.4 Data analysis

To verify hypotheses 1 and 2 the data was analyzed using content analysis and then, the frequency of mention of each word was calculated. The data analysis process ensured the validity and reliability of the study (34). All valid words elicited by the sample were included for data analysis (35). To analyze the content, we created a categorization using the following criteria: comprehensiveness, mutual exclusion, consistency, relevance, objectivity and loyalty, and productivity (36). In this study, we decided not to perform any coding before the data collection, to obtain the codes directly from the data (inductive method). Therefore, following Anguera (37), the starting point was the development of a repertoire or list of behaviors, level of knowledge, etc. carried out in oncology nutrition research. The procedure followed was that of analogical and progressive classification, in which the definition of the category is made at the end of the procedure. Then, we proceeded bidirectionally from the data to the theory and from the theory to the data, deductively and inductively. Later, we followed Corrales et al. (26) purpose of analyzing data (features associated with the role of care and health, and social features). A group of experts (Social Psychology, Nursing, Pharmacology, Medicine, Healthy Psychology, Social Work, and Nutrition) gave a provisional name to these similarities, thus generating a provisional taxonomy in which each category was defined. With this categorization, our purpose was to ensure internal homogeneity between the different items classified in each category and external homogeneity between categories (37). Next, we took the list of categories again and assigned each behavior to the provisional groups already made. Subsequently, we analyzed whether there was an adequate degree of homogeneity between the registered words without making modifications. This way, we assured a comprehensive and mutually exclusive system in each category. Three independent judges codified the items in the selected categories. The consistency and reliability of the classification of coding strategies were obtained through agreement between the three judges (38). The Cohen’s Kappa value exceeded 0.83, indicating a high level of agreement (39). To analyze the content of nutrition in patients and caregivers, we created three variables: two categorical and one ordinal. The first variable shows the category chosen by the person to describe the meaning of eating. The second variable analyzes the presence/absence of the category in the meaning of eating. The third variable measures the frequency of occurrence of each of the categories in the meaning of nutrition for each person.

Thereafter, basic descriptive statistical analyses were carried out and the existence of statistical differences between groups (patients and caregivers) was tested by Chi-Square statistic and t-test. All the statistical analyses were calculated using SPSS 28 and Atlas.ti 6.0 software and p-values < 0.05 were regarded as statistically significant.

## 3 Results

### 3.1 Social representation of nutrition

Concerning the analysis of the perception of nutrition, two samples showed to have more terms. In the free association technique, we found 231 and 234 terms by patients and caregivers respectively. That represented an average of 4 associations of patients and caregivers (range 1-9 words). It is important to know the verbal fluency because it is related to semantic knowledge and, therefore, to the clarity of the message and a stable internal mental representation (40).

For the content analysis, the proposal of Corrales et al. (26) was extended to five categories. Five categories were obtained from the content analysis: health/survival/life (e.g., “healthy”), social relationship (e.g., “intimate relationship”), care (e.g., “care role”), foodstuff (e.g., “vegetables”), and others (“anything”) (see **Table 2**). These were used as factors to be analyzed by the SPSS program. The frequency of the category of nutrition demonstrated that the health/survival/life and foodstuff were the most important categories. Another important category was related to social relationship. Non-significant differences (p-Value>0.05) were found in the perception of nutrition elicited by patients and caregivers.

**Table 2:** Categorization of free word association on the meaning of nutrition.

Health, survival and life	Body maintenance (e.g. body maintenance and balance)
	Physiology (e.g. biology except composition or active principles of foods)
	Health (e.g. health)
	Life/growth (e.g. life)
Pleasant social relationships	Interactions (e.g. relations)
	Feelings (e.g. positive or negative feelings)
Affection and care	Caregiving role (e.g. instrumental support)
	Manifestations of affection (e.g. expressions of affection)
Food/meals	Food (e.g. names of consumer foods)
	Food composition and/or quality (e.g. quality of food)
	Elaboration processing (e.g. techniques or utensils for the preparation, processing or maintenance of foods)
Others	Do not meet the criteria of the other categories

**Table 3** shows the frequency of elicitation for each dimension in each group. The frequency of the perception of nutrition by patient and caregivers demonstrated that the health/survival/life and foodstuff were the most important categories. Another important category was related to social relationship. Afterwards we made a comparison of these scores for the two groups. Non-significant differences (p-Value>0.05) were found in the perception of nutrition elicited by patients and caregivers. Patients related it mainly with names of foods or with the terms related to the elaboration and processing of the same and, later, with health, survival, and life. Caregivers related nutrition, above all, to health, survival, or life as well.

**Table 3:** Frequency of words elicited by categories, level of importance, and whether each of the categories was present or not in the meaning of nutrition.

	Patients						Caregivers					
	Frecuency	%	M	SD	Yes	%	Frequency	%	M	SD	Yes	%
Health/survival/life	80	34.63	2.37	1.33	40	70.2	90	36.88	2.53	1.07	45	78.9
Social relationship	38	16.45	1.65	1.11	19	33.3	33	13.52	1.70	1.11	22	38.6
Care	11	4.76	1.18	0.38	11	19.3	19	7.79	1.26	0.55	13	22.8
Foodstuff	98	42.42	2.61	1.66	38	66.7	88	36.06	2.51	1.61	37	64.9
Others	4	1.73	1.07	0.32	–	–	4	1.94	1.04	0.19	–	–

SD, Standard Desviation; M, Mean.

Then, the perception of nutrition in the free association gave similar data in the two groups of the sample. However, patients related it mainly with names of foods or with the terms related to the elaboration and processing of the same and, later, with health, survival, and life. Caregivers related nutrition, above all, to health, survival, or life as well. This link between diet and care could be due to knowledge and experience about cancer and the impact of nutrition on palliative care for patients and their caregivers. To analyze the perception of each of the groups in more detail, we proceeded to study the benefits and uses of nutrition, as a form of care, as a key to survival, and as a perception of control of cancer. Finally, it was analyzed whether each of the categories was present or not in the meaning of nutrition. As can be seen in the table, the only category that is present in the social representation of nutrition is the one linked to health, survival, and life. Then, the social representation of food has a central part common to patients and caregivers that links it to health, survival, and the importance of food and diet. To analyze the perception of each of the groups in more detail, we proceeded to study the benefits and uses of nutrition, as a form of care, as a key to survival, and as a perception of control of cancer.

### 3.2 Perception of the benefits and uses of nutrition

The same categories of content-analysis were used to analyse the associations proposed regarding the benefits of nutrition in each group (see **Table 4**). A total of 158 terms were revealed over the benefits of nutrition (range 1-4 words) and 168 overuses (range 1-5 words). Among the most frequently used terms were the following: “health” and “life” (patients= 87.95%; caregivers= 84%). However, when the benefits of nutrition were compared, the following differences were revealed: caregivers (χ²=10.007; p<0.01) reflected more social benefit than PCP. The comparison of the categories of uses between groups of subjects showed significant differences (see **Table 5**); Caregivers (16%) had higher scores (χ²=5.361; p<0.05) in social uses of nutrition than patients (8.43%). However, most words were in health/survival/life. In the uses of nutrition category, health/survival/life was the category with the highest score (patients= 83.53%; caregivers= 69.88%). There were significance differences in uses between patients and their caregivers in social relationships. Caregivers had higher scores (patients= 16.47%; caregivers= 25.30%). Then, the perception of nutrition will have significant differences in benefits and uses between the two groups (patients and caregivers).

**Table 4:** Categories elicited by patients and caregivers about benefits of nutrition.

Categories	Patients		Caregivers	
	Frecuency	%	Frecuency	%
Helath/survival/life	73	87.95	63	84.00
Social relationship	7	8.43	12	16.00
Care	0	0	0	0
Foodshtuff	0	0	0	0
Others	3	3.62	0	0

**Table 5:** Categories of patients and caregivers about uses of nutrition.

Categories	Patients		Caregivers	
	Frecuency	%	Frecuency	%
Health/survival/life	71	83.53	58	69.88
Social relationship	14	16.47	21	25.30
Care	0	0	2	2.41
Foodshtuff	0	0	2	2.41
Others	0	0	0	0

### 3.3 Nutrition as an elementary form of care

In the analysis of the perception of nutrition, we showed the importance of nutrition in care. Then we analyzed the relationship between care and nutrition. We asked caregivers to evaluate certain behaviors regarding care (see **Table 6**). One form of care is nutrition. We structured the answers in instrumental, emotional, and nutritional support). A total of 430 answers were revealed. The highest percentages in the responses were given as emotional support (patients= 37.19%; caregivers= 41.56%); however, there were higher percentages of response in both instrumental (patients= 35.17%; caregivers= 35.93%) and emotional type of care (patients= 25.63%; caregivers= 17.32%). Results showed terms referring to the preparation of food. Other words referred to some type of help to eat foods (e.g. “give her breakfast or lunch”). Other terms were, for example, “suffer if you see that I do not eat well”, “ask what you want to eat” or “insist that you eat”. At the same time, it was the caregivers who manifested a greater number of responses related to satisfaction of the patient’s tastes and showed an intent they increase the intake. It is also necessary to mention that a few of the nutritional cares manifested by the caregivers, had negative connotations (“to force to eat”). Moreover, non-significant differences (p> 0.05) were found in categories elicited by patients and caregivers.

**Table 6:** Elementary forms of care.

	Patients		Caregivers	
	Frequency	%	Frequency	%
Instrumental support	70	35.17	83	35.93
Emotional support	74	37.19	96	41.56
Feeding support	51	25.63	40	17.32
Others	4	2.01	12	5.19

### 3.4 Nutrition as a key to survival of palliative cancer patients

We asked about nutrition as a key to the survival of the patient with one question. 65% of patients and 61.63% of caregivers thought that nutrition was important in his/her survival. To explain their opinion, patients argued some questions (see **Table 7**). Nutrition is a key to survival (patients= 25%; caregivers= 17.44%) and it had a positive influence (patients= 38.75%; caregivers= 38.38%). Patients perceived weight loss as a cause of death and to stop eating as a symptom of near death. Patients, who thought nutrition was not important to the evolution of cancer, reported the importance of a healthy life, treatments, and metastasis (patients= 21.25%; caregivers= 23.26%). Other patients reported the importance of religion (patients= 8.75%; caregivers= 3.49%). Results showed that patients and caregivers thought nutrition influenced the survival of the patient although it is not determinant. Moreover, there were no significant differences (p>0.05) between patients and caregivers. Both believe that nutrition is an important aspect related to survival. However, they do not believe that it is the most important. Then, nutrition will be perceived as important to the survival of the patient.

**Table 7:** Relationship between feeding and illness prognostic.

		Patients		Caregivers	
		Frequency	%	Frequency	%
Influence		52	65	53	61.63
	Survivor	20	25	15	17.44
	Positive but not decisive	31	38.75	33	38.38
	Others	1	1.25	5	5.81
Not influence		28	35	33	38.37
	Not feeling	17	21.25	20	23.26
	Spirituality	7	8.75	3	3.49
	Others	4	5	10	11.62

## 4 Discussion

Advances in cancer treatment have extended the lives of people suffering from this disease. However, poor nutrition on the part of patients who disregard nutritional recommendations can make the person worse, influencing their well-being and quality of life. In fact, nutrition seems to play a supportive role in cancer treatment (27) although it is associated with cultural elements with different meanings (29). This study provides information about the social representation of nutrition as a cultural aspect and a specific aspect of palliative cancer patients and their caregivers. In this sense, palliative care personnel should offer guidance to improve the patient’s well-being to for healthcare personnel. Moreover, between professional tasks and, from ‘patient-centered’ approach and Experience-based co-design, involve identifying the changing needs of the patient and the family, establishing the specific care plan, and acting as a mediator between family and patient to adapt to the end-of-life stage (41). It should be done on a collaborative basis analysing the impact of the disease on the psychological and social aspects of food.

The results showed that patients and their caregivers associated nutrition with foods and cooking, life, survival, and health. In this sense, in this study, they related nutrition with foods and cooking. These findings are in line with previous studies for other cultures (e.g., 42) and palliative patients (e.g. 43). But results are inconsistent. Costalat-Founeau et al. (44) showed that French woman score low in this relationship, but Morlot et al. (45) got high scores. We have found that this relationship is high.

Our results additionally show nutrition associated with life, survival, and health. This is similar to other research on nutrition and food (26). Padrón and Barreto (46) showed association of food with health. Other studies, developed in France and Colombia, provide different data on the strength of this link in terms of age (42). These studies showed the variability of the link between food and health, depending on some cultural or sociodemographic factors. In these, French women with an age and culture similar to Spanish caregivers established a low connection between health and nutrition. Our study has had different results. A possible explanation could be that caregivers are in a situation of illness, which would not be present in the case of women in the French study. Moreover, the benefits that palliative cancer patients and caregivers considered from food were more related to health, survival, or life. They also considered benefits at the level of social relationships or the pleasure that food provided them. In addition, the uses that they made of the nutrition was again within the category of health, survival and life and they reported a benefit and use of nutrition at the relational level.

Patients used food to obtain health. This connection is similar to the data analyzed by some authors in general populations of different geographical countries and cultures (e.g. 46) and palliative disease (e.g. 43). This use may be justified by the association of level of intake with the level of energy and strength (47).

Moreover, nutrition is a cultural phenomenon and in Spain, it is very important because it is associated to family time. The Spanish culture highlights its social role and subjective significance (48). In this way, nutrition is the act of eating and a complex phenomenon that links biological, social, and cultural aspects. For palliative cancer patients it can become a focus of stress and for caregivers’ nutrition can become related to care and affection. For both, nutrition becomes something of a symbolic nature that expresses a type of family relationship (23). Furthermore, although food satisfies physiological needs and calms hunger, it is also a channel of communication of feelings, opinions, values ​​, and identity (49). For Mediterranean culture, nutrition has been linked to shared pleasure; in contrast to the values of the Germanic culture in which the pleasure of nutrition is subordinated to health (42). Some researchers suggest that since the 90s, there has been a shift in the social representations of food in the Mediterranean culture as it was directed more towards the values of the Germanic culture (50). However, the Spanish people mostly indicate that “the food they eat matches the foods they like or fancy” (51, p.15] and the data of the present investigation showed a greater importance of health than of pleasure. This link (food is associated with meeting, conversation, and pleasure) is one of the hallmarks of Mediterranean culture (52).

Social support is part of the care practices that occur in family exchanges. Some authors showed that social support is the key factor in the individual’s ability to cope with a stressful situation such as a disease situation (53). For patients and caregivers, the forms of care were similar and food care (materialized in food preparation) was an important category.

Caregivers were the ones who linked food with care the most. This link is already reflected in the literature, generally associated with the female gender (e.g. 54) and particularly in the case of palliative caregivers (e.g. 55). In some cases, nutrition is considered essential and basic care (56). The link between nutrition and care may be due to its everyday nature when care is associated with sickness. Therefore, nutrition becomes care when the disease appears.

This is reinforced by the influence of nutrition and the prognosis of the disease and its link to survival. Most patients and caregivers, who considered that nutrition influenced the prognosis of the disease, expressed that this influence was positive but not determinant. This could be because of the patient’s low capacity to decide or to maintain control over their life. As such, patients considered it important to decide on their diet for psychological well-being and normality. In this way, the conception of nutrition as an activity without impositions was possible (51) for the patient.

Our study has theoretical and practical implications. First, from what we know, there are few investigations that have focused on the psychological, social, and cultural aspects of food and the different uses of nutrition that are not nutrition. Corrales et al. (26) make a theoretical analysis of it. In this sense, this paper contributes to improving knowledge about the social and psychological sense of nutrition. Second, we do not know of studies that have addressed food as a potential problem between the oncological palliative patient and his/her caregiver in Spanish culture. In our culture, the family structures that serve as a support network for the care of the patient show differences with other cultures. The studies about nutrition as a focus of conflict come from the English culture; in which food and care are conditioned by it (57). In our context, researchers have not taken into account the cultural or social aspect of nutrition or other uses of food; they have only studied the nutritional use of food. Therefore, the repercussions of nutrition have been explored little and our study analyzed it.

At the practical level, holistic care of palliative cancer patients and their families implies assessing all aspects related to the patient’s nutrition, including diet and psychological and social aspects. Health personnel have a main role in the management of nutrition for patients and families. In this sense, this paper contributes to improving knowledge surrounding the perception that is associated with nutrition. This knowledge can allow health professionals to understand the behavior surrounding these topics and adapt the care and interventions to such behavior. In this way, one could intervene to redirect the behavior if it is harmful to the patient or to family relationships.

Currently, diagnosis and intervention in palliative cancer patients focuses mainly on anorexia-cachexia when this syndrome is present. However, even in the cases in which it is present, the aspects related to food or nutrition are considered little in health plans. Thus, in most cases, we lack records on the appetite level or intensity of anorexia. Only the presence or absence of anorexia referred by the patient is recorded (58), which is not always similar to the information provided by their caregiver. Moreover, the patient’s weight loss during illness and this specific situation is not routinely assessed, nor are the emotional repercussions. However, weight loss changes the appearance of a patient and deteriorates their image. This absence of records leads to the absence of interventions and caregivers do not receive guidelines on the palliative patient’s nutrition, which can prevent or reduce stress.

This lack of attention by health personnel towards the perception of nutrition and the stress that can be generated between patient and caregiver is important to palliative care. Nutrition is a key aspect in Spanish care. Several authors highlight the complaints of patients and caregivers about the lack of attention to nutrition and nutritional problems. In addition, they demand continuous and up-to-date nutrition education, as well as support for the family in this issue from the palliative care team (59). All this indicates that anorexia, weight loss and problems related to food, are significant problems for the patient and caregiver. Thus, following the advice of Loeliger et al. (5) of the design of nutrition services, this study allows us to analyze the nutritional needs and experiences of caregivers and to contribute to specific strategies to improve the nutritional information of cancer patients and their care.

Our study had some limitations, and the results should be interpreted with caution. First, the sample size was small and, thus, the power of the results can be affected. It would have been better to have more patients and caregivers. However, the low participation rate of palliative cancer patients and their family caregivers was in line with other studies on this topic (e.g., 55). The high mortality of the sample is similar to other research on this type of patient, which also has lower shares than 50% (60). Nevertheless, future research should replicate this study with larger sample sizes. Second, this study focused on the perception of nutrition of Spanish cancer palliative patients and their caregivers. Consequently, these findings may not be generalizable to other cultures. However, nutrition is a multifactorial phenomenon that links biological, social, and cultural aspects. Hence, future research should conduct studies to investigate this phenomenon, first, in each culture and, second, the regularities in all cultures. Third, the potential role of women as caregivers was not investigated in this study. Some authors show nutrition as a form of care associated with the female gender (55) and in Spain, it is more visible. However, our research is a first step in the analysis of the perception of nutrition. Thus, future research should conduct studies to link gender and the care of palliative cancer patients. Fourth, a limitation of the study is related to the fact that all the data was cross-sectional and gathered from a single source. We wanted to analyze narrative data and use interviews because it is a valuable method to explore perceptions in a natural setting (61). However, future research should also use observational analysis or focus groups.

In this sense, our study makes several contributions to the literature. First, our study improves knowledge because it analyzes the social representations of nutrition of cancer patients in advanced-stage and palliative care and caregivers. Second, on a practical level, palliative care should support families in understanding the perception of nutrition and patient suffering (62). Along with this, the information provided by the patient about their perception of nutrition is the basis of their experience and turns out to be the heart of the design of their care from the patient-centered approach and, more specifically, from the EBCD.

## 5 Conclusion

The aim of this research was to analyze the perception of nutrition in the context of oncological palliative disease; as well as whether nutrition constitutes a potential source of stress, between the palliative cancer patient and his/her caregiver within Spanish culture. This study provides evidence that shows the importance of the perception of nutrition for the well-being of patients and caregivers because it is a form of care. Our findings suggest that the perception of nutrition is linked to life, survival, and health. Cancer, in its advanced stages, changes the patient’s diet, which has an impact on the way they eat and on family and social dynamics. These changes generate negative repercussions for the patient. Therefore, nutrition must be considered as a basis for palliative service interventions. In this sense, our study has implications for health practices. The interventions for the palliative cancer patient and their family should aim to provide comfort and improve the quality of life and include nutrition as content. Thus, knowledge of the influence exerted by food contributes to improving the praxis of health personnel in palliative care and opens future lines of research in the nutritional care of these patients.

## Data Availability

The datasets presented in this study can be found in online repositories. The names of the repository/repositories and accession number(s) can be found below: https://zenodo.org/record/8345048.
